# A Review of Luting Agents

**DOI:** 10.1155/2012/752861

**Published:** 2012-02-22

**Authors:** Cornelis H. Pameijer

**Affiliations:** Department of Reconstructive Sciences, School of Dental Medicine, University of Connecticut, Farmington, CT 06030, USA

## Abstract

Due to the availability of a large number of luting agents (dental cements) proper selection can be a daunting task and is usually based on a practitioner's reliance on experience and preference and less on in depth knowledge of materials that are used for the restoration and luting agent properties. This review aims at presenting an overview of current cements and discusses physical properties, biocompatibility and other properties that make a particular cement the preferred choice depending on the clinical indication. Tables are provided that outline the different properties of the generic classification of cements. It should be noted that no recommendations are made to use a particular commercial cement for a hypothetical clinical situation. The choice is solely the responsibility of the practitioner. The appendix is intended as a guide for the practitioner towards a recommended choice under commonly encountered clinical scenarios. Again, no commercial brands are recommended although the author recognizes that some have better properties than others. Please note that this flowchart strictly presents the author's opinion and is based on research, clinical experience and the literature.

## 1. Introduction

Proper selection of a luting agent is a last important decision in a series of steps that require meticulous execution and will determine the long-term success of fixed restorations. One hundred years ago this decision was easy with the availability of essentially only one luting agent, zinc phosphate cement. Currently, a plethora of luting agents is available. Now the choice of the optimal luting agent can be confusing, even for the most experienced clinician. Restorations of metal, porcelain fused to metal, low-and high-strength ceramics, full or partial coverage, require a prudent approach and the proper cement selection should be based on knowledge of physical properties, biological properties and other attributes of both restorative materials and luting agents. This paper aims at providing an overview of currently available luting agents (cements) and discusses their advantages and disadvantages. Emphasis has been placed on composition, biocompatibility, physical properties, clinical indications, and clinical performance. A wide range of formulations has been developed over the last 40 years, but here emphasis has been placed on the contemporary most frequently used ones, whether used for luting or bonding.

## 2. Classification of Cements

Cements can be classified as follows:

1. liners and bases;
2. temporary (provisional) cements;
3. permanent cements.

### 2.1. Liners and Bases

Preference seems to be given by the dental profession to visible light curing materials, in particular resin-modified glass ionomer (RMGI) cements (sometimes also referred to as resin reinforced glass ionomer (RRGI), when there is a need for a base or a liner. The reason is based on simplicity and on the fast setting characteristics of light curing materials as well as the possibility of subsequently etching them in order to establish strong adhesive bonds with dentin bonding agents. Furthermore, they adhere well to unetched hard tissue and exhibit sustained fluoride release.

### 2.2. Provisional Cements

Provisional cements can be eugenol, noneugenol, resin, or polycarboxylate based. Caution has to be exercised when using eugenol-containing cements as the eugenol can contaminate the preparation. This can inhibit the polymerization of certain resin composites subsequently used as permanent restorative filling material [1].

Eugenol-containing temporary cements that are used prior to indirect bonding restorations reduce the bond strength of both total- and self-etching adhesive systems to dentin [2]. It is therefore advisable to use noneugenol temporary cements. In another report, however, no difference in bond strengths was observed when using eugenol-free and eugenol-containing provisional cements followed by self-adhesive resin cements [3].

Most subsequent publications report on a reduced bond strength of luting agents when eugenol-containing temporary cements are used [4, 5]. Nevertheless, the application of any temporary cement, whether eugenol-containing or not, contaminates the dentin, which will interfere with adhesion.

### 2.3. Permanent Cements

**Figure 1:**
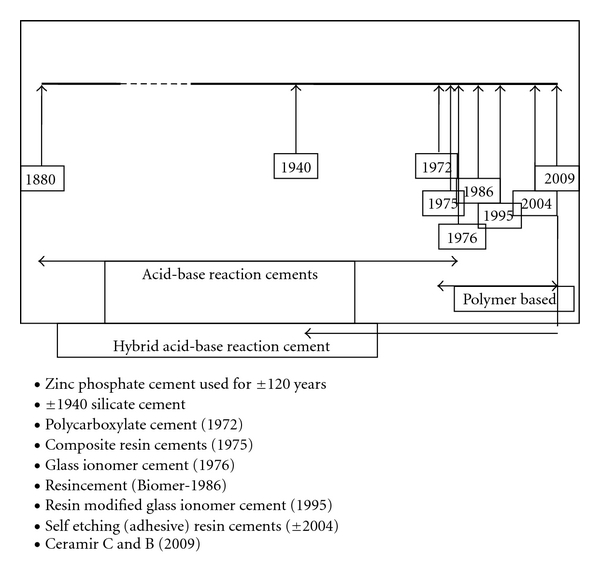
An overview of the chronological development of luting agents starting around 1880 until today. The last 30–40 years have witnessed the development of new cement systems and a large number of cements have become available. It was not until 2009 that a paradigm shift took place and a hybrid acid-base reaction cement was introduced, which offered physical and other properties that not only differed from the polymer-based luting agents but also matched them. +1880—zinc phosphate cement, +1940—silicate cement*, 1972—polycarboxylate cement, +1975—composite resin cements, 1976—glass ionomer cement, 1986—resin cement, +1995—resin-modified glass ionomer cement, +2004—self-etching (adhesive) resin cements, 2009—hybrid-acid-base reaction cement, (*the designation silicate cement is a misnomer as it was a restorative material for Cl III and Cl V restorations).

Figure 1 shows the chronological development of luting agents from the late 1800 hundreds to today. It is significant in that for almost 100 years only zinc phosphate cement was available, which is still being considered the “gold” standard.

With the introduction of cast restorations in the late 1880s, the need for a luting agent or dental cement for crowns and small bridges was readily recognized by the dental profession. The Dental Cosmos reported (in the late 1800s), a technique for the fabrication of a 4-unit pin ledge bridge (Finley), which required cement for fixation. While gold shell crowns were introduced around 1883 it was not until 1907 that Taggert introduced cast crowns by means of the lost wax technique. Around 1879, zinc phosphate cement was introduced and although the formulation has been refined during more than a century of use, it is a luting agent that has consistently been successful in clinical practice and even today is still considered the “gold” standard. With the exception of silicate cement in the 1940s few new cements were introduced until around 1970. The word silicate cement, however, is a misnomer as it was not a luting agent. It was used for anterior Cl III and Cl V esthetic restorations.

## 3. Zinc Phosphate Cement

The cement comes as a powder and liquid and is classified as an acid-base reaction cement. The basic constituent of the powder is zinc oxide. Magnesium oxide is used as a modifier (±10%) while other oxides such as bismuth and silica may be present.

The liquid is essentially composed of phosphoric acid, water, aluminum phosphate, and sometimes zinc phosphate. The water content is approximately 33 ± 5% and is an important factor as it controls the rate and type of powder/liquid reaction [6].

When the powder reacts with the liquid a considerable amount of heat is generated (exothermic reaction) and when the mixing is complete the cement reaches a pH of 3.5. Since the cement is placed on and in prepared teeth when it is in a “wet consistency” and not all the liquid has reacted with the powder, unreacted phosphoric acid liquid with a low pH ±1.5 comes in contact with the preparation and causes an immediate (within 5 s) dissolution of the smear layer and smear plugs. Since cementation can cause a considerable amount of hydraulic pressure, the unreacted acid is pressed in the dentinal tubules and, depending on the remaining dentin thickness (RDT), the distance from the floor of the preparation to the pulp, can cause greater or less irritation to the pulp. Therefore, the pulp has to cope with not only heat but low acidity as well. The greater the RDT, the more beneficial the buffering action of the fluid in the dentinal tubules is and the less the effect of the acid. Furthermore, a greater RDT also diminishes the thermal effect. When fully reacted, the set cement reaches a pH = 6.7 after 24 hours. Postcementation hypersensitivity is indeed a frequently occurring clinical problem, which either resolves over time or may result in the need for endodontic treatment. If it resolves, it is through the protective action of secretion of secondary dentin by the odontoblasts, which increases the RDT. This however, does not start in humans until 3 weeks after the insult has taken place and deposition of secondary dentin occurs in microns per day [7]. If the irritation cannot be handled by the body, the pulp becomes necrotic, which then requires root canal treatment. Therefore, although the set luting material may be biocompatible, postcementation discomfort is a known unfavorable side effect when using this cement. Attempts at blocking access of the unreacted phosphoric acid to the dentinal tubules have been made in the form of a varnish (Copalite). Unfortunately, Copalite can reduce the retention of the restoration by as much as 50% [8].

## 4. Zinc Polycarboxylate Cement

Polycarboxylate cement is also an acid-base reaction cement. The powder is composed of mainly zinc oxide, magnesium oxide, bismuth, and aluminum oxide. It may also contain stannous fluoride, which increases strength. The liquid is composed of an aqueous solution of polyacrylic acid or a copolymer of acrylic acid and other unsaturated carboxylic acids. Fluoride release by the cement is a small fraction (15–20%) of that released from materials such as silicophosphate and glass ionomer cements.

When mixed at the recommended P/L ratio the final mix appears more viscous than zinc phosphate cement. However, this can be offset by vibratory action during seating yielding a film thickness of ±25 *μ*m. At no time should the amount of liquid be increased, as it will adversely affect the compressive strength, which at 55 MPa is already lower than that of zinc phosphate cement. Biological properties of polycarboxylate cement are quite favorable and the cement causes little or no irritation to the pulp, even at a remaining dentin thickness of 0.2 mm (Unpublished data). It is believed that the long molecular chains of the polyacrylic acid prevent penetration into the dentinal tubules. It is of interest to note that both zinc phosphate and polycarboxylate cements have a pH of about 3.5 immediately after mixing. Currently polycarboxylate cements are mostly used for long-term temporary cementation.

Polycarboxylate and glass ionomer cements exhibit a property that is called chelation, which is the ability to bond to the Ca ions.

## 5. Glass Ionomer Cement

Glass ionomer cements (GICs) were invented in the late 1960s in the laboratory of the Government Chemist in Great Britain and were first reported on by Wilson and Kent in 1971 [9]). GICs set by means of chelation as a result of an acid-base reaction. They strongly adhere to enamel and to some extent to dentin and release fluoride. Initially used as a restorative material, GI further evolved into a luting agent, which is now the predominant application of this class of material.

The powder consists of aluminosilicates with high fluoride content. The material is formed by the fusion of quartz, alumina, cryolite, fluortite, aluminum trifluoride, and aluminum phosphate at temperatures of 1100–1300°C. This glass frit is cooled to a dull glow and quenched in water. It is subsequently ground into 45 *μ*m particles.

The liquid is composed of polyacrylic acid and tartaric acid, the latter to accelerate the setting reaction. The reaction of the powder with the liquid causes decomposition, migration, gelation, postsetting hardening and further slow maturation. The polyacrylic acid reacts with the outer surface of the particles resulting in release of calcium, aluminum, and fluoride ions. When a sufficient amount of metal ions has been released, gelation occurs, and hardening continues for about 24 hours [9].

GIC display a relatively low curing shrinkage; within the first 10 minutes 40–50% of shrinkage has occurred.

However, with the use of GIC as a luting agent, frequent postcementation sensitivity has been reported. The then accepted ANSI/ADA Specification 41, Recommended Standard Practices for Biological Evaluation of Dental Materials stipulated that luting agents should be tested for pulp reaction in primates by passively inserting a heavier than luting consistency mix in Class V restorations in primates. Indeed the results of these tests demonstrated that the cement was biocompatible and nonirritating [10]. In a subsequent study, also in primates, crowns were cemented adhering to a clinically more relevant cementation protocol, with a cement mix that had a normal luting consistency [11].

In this study hydraulic pressure generated during cementation and the resulting penetration of unreacted acid into the dentinal tubules was responsible for the true postcementation reaction of the pulp under clinical conditions. It was clearly demonstrated that, depending on the RDT, GIC caused pulpal inflammation which, rather than subsiding over time, increased in severity. It was this study that resulted in a change in protocol in the ANSI/ADA Specification 41 (2005) [12], which now calls for a pressure insertion technique. Rather than using a laborious indirect technique and cementing all metal cast crowns as was done in the aforementioned study, Cl V composite resin inlays are fabricated and cemented with the cement to be tested. With the use of this technique, hydraulic pressure is generated that is similar to complete crown cementation. In addition, the Cl V inlays are usually closer to the pulp than crown preparations and therefore result in a more reliable biocompatibility reaction.

## 6. Resin Cements

As an alternative to acid-base reaction cements, resin cements were introduced in the mid-1980s, these materials have a setting reaction based on polymerization. Resin cements are polymers to which a filler has been added as well as fluoride. Cement film thickness is not favorable for some materials, for example, C & B Metabond (Parkell Inc.) with a film thickness > 100 *μ*m, while others have a reported film thickness of 9 *μ*m, for example, Permalute (Ultradent Products Inc). One of the first resin cements was marketed by Dentsply/Caulk under the name Biomer, around 1987. In two clinical studies by Pameijer (unpublished data), the cement performed well over a one-year period of evaluation. However, over time polymer degradation occurred due to hydrolysis, while a lack of bonding to enamel and dentin made the cement unsuitable as a stand-alone luting agent, leading to leakage and failure of the restoration. Additionally, incomplete polymerization can lead to irritation of the pulp by unreacted monomers.

In combination with a dentin bonding agent, however, many resin cements have superior properties and are frequently used for the cementation (bonding) of porcelain laminate veneers. The concept of a “monobloc” described in endodontics [13] applies here as well. A combination bonding agent that bonds to tooth structure and a resin cement that adheres to the bonding agent and to silane treated porcelain follows the same principles. Nevertheless, there is a reluctance on the part of practitioners to do a “total etch” of complete crown preparations, which is a required step for many bonding agents. Even the self-etching dentin bonding agents are not ideal because of concerns for postoperative sensitivity.

## 7. Resin-Modified Glass Ionomer (RMGI) Cements

The RMGI or RRGI (resin-reinforced glass ionomer) cements are indicated for the luting of crowns and bridges, as well as inlay and onlay restorations. They are essentially hybrid formulations of resin and glass ionomer components. The RMGI cements are relatively easy to handle and are suitable for routine application with metal-based crown and bridgework. However, their use is limited when adhesively cementing ceramics with smooth, nonretentive surfaces. Adhesion to tooth structure is not strong with these materials. Additionally, some early formulations have displayed excess water sorption, causing swelling frequently resulting in ceramic fracture. Commercial examples of the RMGI cements are: RelyX Luting, RelyX Luting Plus (3 M/ESPE), Fuji Plus (GC) and UltraCem RRGI Luting Cement.

In a recent article, the biological effects of resin-modified glass-ionomer cements as used in clinical dentistry were described, and the literature reviewed on this topic [14]. Information on resin-modified glass ionomers and on 2-hydroxyethyl methacrylate (HEMA), the most damaging substance released by these materials, was collected from over 50 published papers. These were mainly identified through Scopus. It is known that HEMA is released from these materials, which has a variety of damaging biological properties, ranging from pulpal inflammation to allergic contact dermatitis. These are therefore potential hazards from resin-modified glass ionomers. However, clinical results with these materials that have been reported to date are generally positive. According to the above authors, RMGIs cannot be considered biocompatible to nearly the same extent as conventional glass-ionomers. Care needs to be taken with regard to their use in dentistry and, in particular, dental personnel may be at risk from adverse effects such as contact dermatitis and other immunological responses. Interestingly, RMGIs have a better clinical track record than glass ionomer cements.

In general few complaints have been reported about postoperative cementation hypersensitivity. Yet, RMGIs are in the category of resin cements and water sorption and degradation through hydrolysis are negative features that should not be ignored or underestimated.

In spite of the numerous research methodologies that are at our disposal conflicting results are frequently reported, either using the same technique and tests on the same materials, or using different techniques and testing the same materials. RMGIs as shown above are such an example. While controversial data has been generated, successful clinical use seems to contradict these findings.

## 8. Adhesive Resin Cements

The poor adhesive properties of the RMGIs have led to further development of resin-based luting agents, which have resulted in the introduction of adhesive resin cements. These cements do not require pretreatment and bonding agents to maximize their performance. In order for these cements to be self-adhesive, new monomers, filler and initiator technology were created. Examples of these materials are: MaxCem (Kerr), RelyX Unicem (3 M/ESPE), Breeze (Pentron), Embrace Wet Bond (Pulpdent Corporation) to name a few. These cements enjoy great popularity as they have universal applications. As pointed out before under resin and RMGI cements, polymer degradation over time is still an issue. Matrix metalloproteinases (MMPs) are fossilized within mineralized dentin and can be released and activated during bonding [15]. These endogenous collagenolytic enzymes are on the collagen fibers and needed for bonding and their slow degrading enzymatic action is beyond the control of even the most meticulous clinician. Reports have appeared that recommend pretreatment of the dentin with 2.0% chlorhexidine gluconate with a pH of 6.0, which prevents the action of the endogenous enzymes [16].

## 9. Hybrid-Acid-Based CaAl/Glass Ionomer

Only one formulation is presently known that is based on calcium aluminate/glass ionomer. Ceramir C&B (Doxa Dental AB, Uppsala, Sweden) is a new dental luting agent intended for permanent cementation of crowns and bridges, gold inlays and onlays, prefabricated metal, and cast post and cores and all-zirconia or all-alumina crowns. The cement is a water-based hybrid composition comprising of calcium aluminate and glass ionomer components that is mixed with distilled water. The material has been demonstrated to be bioactive [17]. The setting mechanism of Ceramir C&B is a combination of a glass ionomer reaction and an acid-base reaction of the type occurring in hydraulic cements. The incorporation of the calcium aluminate component provides several unique properties compared to conventional GIC's. There are several features that strongly contribute to the biocompatibility profile of the material. These include the fact that after setting, the material is slightly acidic, pH ~4. After 1 h, the pH is already neutral and after 3-4 hrs it reaches a basic pH of ~8.5. This means that the fully hardened material is basic and stays basic throughout its service. This basic pH is the most important prerequisite for the material to be bioactive, that is, creating apatite on its surface when in contact with phosphate-containing solutions [17]. The apatite forms during hardening but its formation continues when the hardened material is in contact with phosphate solutions. The basic pH is also an important factor in the biocompatibility profile of the material. Additionally, the material produces an excess of Ca^2+^ ions, which also contributes to its bioactivity. The incorporation of calcium aluminate fixes the GIC structure and hinders the ionomer glass from continuously leaking over time. Ceramir C&B has an initial fluoride release comparable to a glass ionomer, although the release tapers off over time. Unique properties such as apatite formation and remineralization develop quickly and continue to be active.

## 10. Pulpal Reactions

Ultimately, a postcementation pulpal reaction under clinical conditions is dependent on three factors:

1. composition of the cement. Postoperative hypersensitivity for most cements can be problematic and is based on their chemistry, while only a few do not present a problem;
2. the RDT—the larger the RDT the less risk of pulp irritation due to the greater buffering capacity of the fluid in the dentinal tubules;
3. time elapsed from preparation to moment of cementation—the longer this period, the better the pulp is able to recover from the trauma of preparation and therefore can tolerate a subsequent irritation better.

## 11. Biocompatibility

Luting agents for permanent cementation of crown and bridge restorations have to meet many requirements before they can safely be used in humans. The ANSI/ADA Recommended Standard Practices for Biological Evaluation of Dental Materials, Specification 41 (2005) [12], and the ISO 7405 provide a road map outlining tests that are required in order to meet these requirements. Physical properties such as hardness, flexural strength, and solubility are extremely important but if the material lacks biocompatibility, excellent physical properties are meaningless. For practitioner and patient alike, a luting agent that causes no postcementation hypersensitivity is highly desirable. Dentistry is still perceived by many, as being “a painful experience” and every effort should be made on the part of the dentist to make the treatment as comfortable as possible. One such step is the final cementation of a fixed crown and bridge work, whether a single unit or a bridge. A restoration may be esthetically pleasing and functional at the time of cementation, but a sequel of postcementation hypersensitivity can generate questions from the patient as to the success of the treatment, time from the practitioner to address the problem, and possible complications that require further treatment. Extra visits may be required, all of which constitute a loss of time and money not only for the practitioner, but also for the patient.

Although zinc phosphate cement is still the “gold” standard, advances in luting agents over the last 30 years have produced new luting agents, which most likely will eventually replace zinc phosphate cement altogether. If we look at the three acid-base reaction cements, zinc phosphate, polycarboxylate and glass ionomer cement and compare them to the hybrid-acid-base reaction cement, two of the three cements (zinc phosphate and glass ionomer cements) have well recognized postcementation hypersensitivity problems. This has frequently resulted in the need for root canal treatment after permanent cementation of the fixed unit. Typical complaints of a patient are sensitivity to hot and cold and chewing. Assuming that the occlusion is not a causative factor, the only explanation is irritation caused by the cement. Clearly, if the patient was comfortable during the interim with a provisional restoration the problems point towards the irritation caused by the permanent cement. Mostly the pain will subside, more so with zinc phosphate cement than glass ionomers, but this may take weeks or longer, and the practitioner can only guess at the ultimate outcome. *In vivo* research has shown that indeed after cementation with zinc phosphate cement and glass ionomers causes pulpal irritation, which would explain the complaints from patients [11].

RMGIs also have a record of occasional postcementation hypersensitivity due to their questionable biocompatibility [14]. In particular, unreacted monomers are highly toxic and irritating.

The resin cements and self adhesive resin cements have a good track record, although there are few, if any, reports that support their biocompatibility.

Little clinical data are available on self-adhesive cements. Empirical data suggest that they are tolerated by the pulp, perhaps based on the change in acidity upon complete setting.

The many properties that are exhibited by luting agents are summarized in Tables 1 and 2.

Tables 1 and 2 clearly show the differences between the various generic cements. It is therefore important that the practitioner is familiar not only with the composition and properties of the luting/bonding agent, but also with the composition of the restoration to be cemented.

A separate flow chart is being presented in the appendix, which serves as a guide for the practitioner in the selection of a final luting agent. Hypothetical clinical situations are being presented that can be cross-referenced with a choice of a generic cement. The chart is based on clinical observations, research, and the literature.

## 12. Concluding Remark

The choice of an appropriate luting agent (cement) for final cementation of fixed crown and bridge units needs careful consideration as the ultimate success to a large extent depends on the correct choice.

## Figures and Tables

**Table 1:** This Table compares properties of the various generic cements. (Biocomp: biocompatibility; Integr: integration, Oxy inh layer: oxygen inhibited layer, RRGI: resin reinforced glass ionomer).

Cement	Universal	Retention	Biocomp	Sensitivity	Integr	Self-Etch	Self-Seal	Bioactive	Oxy inh layer
Zinc Phosph	No	Low/med	*	Yes	No	No	No	No	No
Polycarb	No	Low	****	No	No	No	No	No	No
Glass ion	No	Medium	***	Yes	No	No	No	No	No
Resin	No	Medium	***	No	No	No	No	No	Yes
RRGI	Yes	Med/high	***	?	No	No	No	No	yes
Self-etch resin cement	Yes	High	****	?	?	Yes	No	No	Yes
Hybrid CaAl/GI	Yes	High	*****	No	Yes	Yes	Yes	Yes	No

**Table 2:** Comparison of additional properties of the various generic cements.

Cement	Nano crystals	Hydroxy apatite	Hydrolysis	Water sorption	Resin-based	Mineralizing	F-release
Zinc Phosph	No	No	No	No	No	No	No
Polycarb	No	No	No	No	No	No	No
Glass ion	No	No	No	No	No	Yes	Yes
Resin	No	No	Yes	No	Yes	No	Yes
RRGI	No	No	Yes	Yes	Yes	No	Yes
Self-etch resin cement	No	No	Yes	Yes	Yes	No	Yes
Hybrid CaAl/GI	Yes	Yes	No	No	No	Yes	Yes

**Table 3:** Clinical indications for use of luting agents.

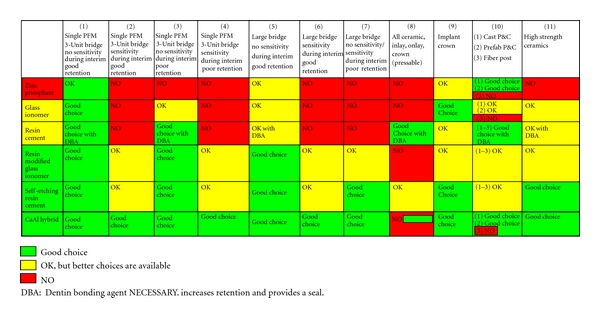

## Appendix

See Table 3.
